# Computational Prediction and Validation of Tumor-Associated Neoantigens

**DOI:** 10.3389/fimmu.2020.00027

**Published:** 2020-01-24

**Authors:** Vladimir Roudko, Benjamin Greenbaum, Nina Bhardwaj

**Affiliations:** ^1^Department of Hematology and Medical Oncology, Icahn School of Medicine at Mount Sinai Hospital, New York, NY, United States; ^2^Center for Computational Immunology, Tisch Cancer Institute, Icahn School of Medicine at Mount Sinai Hospital, New York, NY, United States; ^3^Department of Pathology, Icahn School of Medicine at Mount Sinai Hospital, New York, NY, United States; ^4^Department of Oncological Sciences, Icahn School of Medicine at Mount Sinai Hospital, New York, NY, United States

**Keywords:** neoantigen, TCR, WES, HLA-allele, MHC-I epitope

## Abstract

Tumor progression is typically accompanied by an accumulation of driver and passenger somatic mutations. A handful of those mutations occur in protein coding genes which introduce non-synonymous polymorphisms. Certain substitutions may give rise to novel, tumor-associated antigens or neoantigens, presentable by cancer cells to the host adaptive immune system. As antigen recognition is the core of an effective immune response, the identification of patient tumor specific antigens derived from transformed cells is of importance for immunotherapeutic approaches. Recent technological advances in DNA sequencing of tumor genomes, advances in gene expression analysis, algorithm development for antigen predictions and methods for T-cell receptor (TCR) repertoire sequencing have facilitated the selection of candidate immunogenic neoantigens. In this regard, multiple research groups have reported encouraging results of neoantigen-based cancer vaccines that generate tumor antigen specific immune responses, both in mouse models and clinical trials. Additionally, both the quantity and quality of neoantigens has been shown to have predictive value for clinical outcomes in checkpoint-blockade immunotherapy in certain tumor types. Neoantigen recognition by vaccination or through adoptive T cell therapy may have unprecedented potential to advance cancer immunotherapy in combination with other approaches. In our review we discuss three parameters regarding neoantigens: computational methods for epitope prediction, experimental methods for epitope immunogenicity validation and future directions for improvement of those methods. Within each section, we will describe the advantages and limitations of existing methods as well as highlight pressing fundamental problems to be addressed.

## Introduction

Successful targeting of immune checkpoints including cytotoxic T lymphocyte-associated protein 4 (CTLA-4) and programmed cell death protein 1 (PD-1) has achieved durable regressions in a wide range of human cancers (referred to as checkpoint blockade). They include melanoma (1, 2), renal cell carcinoma (3), lung (4), bladder (5), and ovarian cancers (6), and microsatellite unstable malignancies (7, 8). Despite different mechanisms of action, both approaches have resulted in the activation and proliferation of tumor-reactive T cells (9). T cells recognize peptides presented on the major histocompatibility complex (MHC) of tumor cells. Tumor specific antigens which arise due to mutations in coding regions are collectively referred to as “neoantigens.” Neoantigens have a diversity of properties. They can differ from their wild type sequences by SNV, relative expression levels in the tumor, MHC affinity, differential recognition by T-cell receptors (TCRs) and elicitation of heightened cytotoxic and cytokine responses. Theoretically, T cells recognizing neoantigens may have not been deleted or tolerized so they have the potential to become primed. Moreover, unlike tumor-associated antigens (TAA) that are shared between tumor cells and normal tissue (e.g., Melan A/MART-1) neoantigens have a selective potential to elicit tumor exclusive T cell responses which makes them key elements for inclusion in cancer vaccines and as the basis for adoptive T cell transfer approaches (10–14). Indeed, initial attempts to target overexpressed TAA have met limited success in clinical trials potentially due to central and peripheral tolerance mechanisms which removes high-affinity TCRs that would otherwise potently recognize these TAA (15, 16). Unleashing immune responses against tumor-specific clonal mutations can achieve tumor regression through recognition by antigen-specific T cells (17–22). Furthermore, as a tumor's mutational landscape evolves with ongoing immunotherapy, the immune system may accommodate by changing the specificity of infiltrating T cell clones (23–25). Efficient approaches to identify and characterize immunogenic tumor neoantigens are central for these types of therapies.

**Figure 1:**
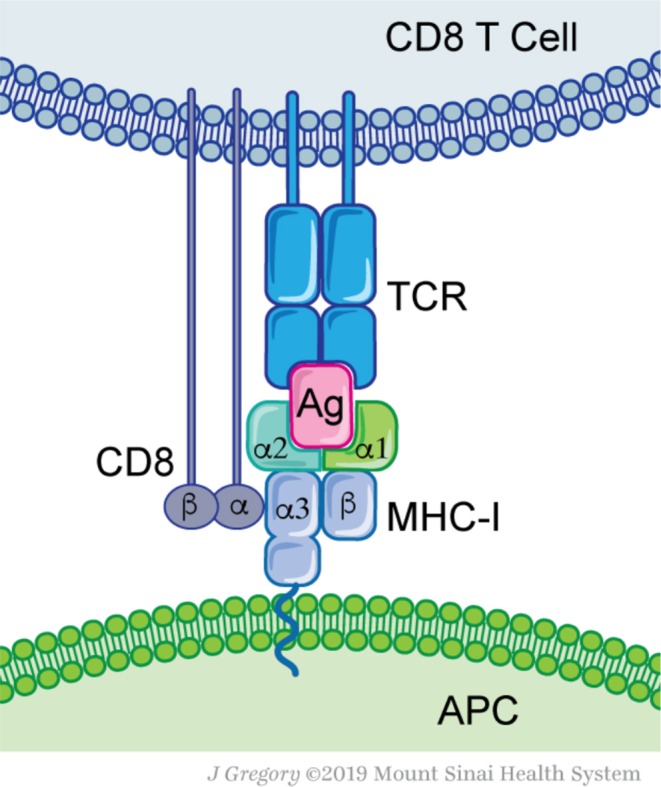
Molecular basis for antigen recognition. Antigen-presenting cells (APC) express MHC-I complex that contains an antigenic peptide (Ag) with its groove. MHC-I consist of two proteins, a conserved β_2_-microglobulin and a variable α-chain. The MHC-I-Ag complex is recognized by the T-cell receptor (TCR). Each TCR defines a clonal T cell population. Additional interactions, such as the CD8 protein—MHC-I, are not essential for Ag recognition, but are required for efficient T cell activation.

Thus, far, MHC-I affinity is the only parameter which can be predicted with some reliability using neoantigen peptide and patient HLA allele sequences *in silico*, by using several computational tools. Our group recently proposed the concept of “neoantigen quality” (26, 27). This concept combines biophysical, chemical and computationally inferred properties of a neoantigen that make it more likely to induce a productive immune response against the tumor. These properties may include affinity of a neoantigen to MHC, avidity of the peptide-MHC complex to the recognizing TCR, type of T cells responding to the neoantigen and sequence similarity to known highly immunogenic epitopes (Figure 1). Recent studies from our group have shown that this parameter is a critical aspect in segregating responders to checkpoint therapy, but is not usually considered in algorithms of neoantigen prediction.

T cells are primed by antigen presenting cells (APC) that have taken up tumor antigens and processed them into smaller peptides that are eventually presented on MHC class I and II molecules (Figure 1). Intracellularly, antigens arise from proteins targeted for degradation by the 80S proteasome. Peptides of 9-12 amino acid residues in length are transported from the cytosol by specialized protein machinery (transporter associated with antigen presentation, TAP) and loaded on MHC-I molecules within the endoplasmic reticulum (28–30). Alternatively, antigens can arise from extracellular sources; captured necrotic or apoptotic cells and other vesicles that are cross-presented on professional APC such as dendritic cells (DC) (30, 31). As a tumor grows, tissue resident and migratory DC subsets capture tumor cell debris and convey them to draining lymph nodes (32–34). There, APC prime naïve T cells and educate them to recognize the harvested antigens (35, 36). Depending on the APC subset, nature of antigen and type of processing pathway, different responses can be achieved, either CD4+ T cell responses (Th1, Th2, Th17, and Treg) or cytotoxic CD8+ T cell responses (37, 38). The majority of APC prime naïve CD4+ T cells through MHC-II presented peptides, while the cross-presenting XCR1+ DC subset uniquely primes naïve CD8+ T cells (39, 40). The latter appear to be essential for successful immunotherapy regimens (41–43). After priming, reactive and expanded T cells can infiltrate the cancer site and eliminate these cells. Overall, proper antigen selection, processing, and T cell priming are at the heart of successful immune responses.

With the recognition that neoantigens can be a significant pool of tumor derived antigens depending upon the underlying mutational status of the tumor, the field has turned its attention to developing and optimizing neoantigen targeted immune therapies. There are generally two approaches: neoantigen vaccines and neoantigen targeted adoptive T cell therapies. Several clinical trials have been completed and/or are currently ongoing to enhance tumor-specific responses through neoantigen vaccination to induce expansion of neoantigen-specific CD4+ and CD8+ T cells. Vaccination techniques employ different neoantigen formulations such as peptides (13, 44, 45) combined with different adjuvants (46–49), mRNA (50, 51), DNA or as expressed in viral or bacterial vectors. Another interesting approach targets neoantigens to specific receptors expressed specifically by the cross-presenting APC cDC1. Here antigens are fused in frame to antibodies targeting the XCR1 receptor (52). Thus, far, vaccine-based approaches have demonstrated successful immunization of patients (53, 54), although CD4+ as opposed to CD8+ T cells are preferentially generated. Cell-based vaccination is also under investigation. *In vitro* expanded, neoantigen-pulsed dendritic cells have been evaluated for autologous injection in patients (46, 55–58) confirming immunogenicity (57, 58). Another approach focuses on the adoptive T cell transfer of expanded T cells purified from the patient's tumor or peripheral blood mononuclear cells (PBMC) either non-specifically or through selection *in vitro*. Some strategies have successfully utilized neoantigen-specific CD4+ and CD8+ cytotoxic T cells to eradicate solid tumors (59, 60). In another example, autologous T cell transfer of CD8+ T cells, specific to clonal neoantigens derived from cancer driver mutations, e.g., KRAS, has led to nearly complete tumor regression (61).

Mutation burden, neoantigen burden and quality have been demonstrated to be predictive for outcome of checkpoint blockade (26, 62–70). A few studies have highlighted the importance of neoantigens in shaping tumor evolution during immunotherapy with antibodies that target checkpoint molecules such as CTLA-4 and PD-1 (71, 72). However, neoantigen prediction approaches are not aligned with some utilizing solely gene expression (73, 74) or combining transcriptomics with genomics (75). The successful characterization of immunogenic neoantigens is critical to optimizing approaches that target these key epitopes. In this review we critically discuss current tools and methods for their selection (76).

## The Landscape of Neoantigens

**Figure 2:**
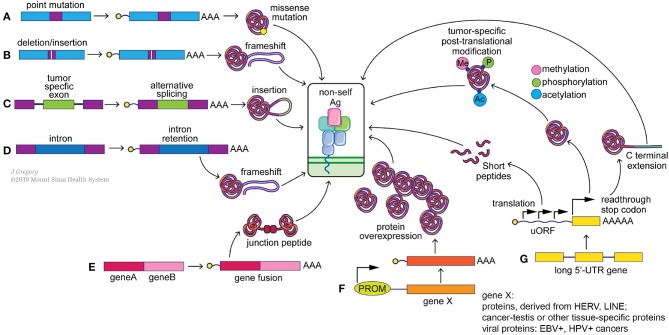
Potential sources of “non-self” tumor neoantigens. Genomic alterations such as point mutations **(A)**, indels **(B)** and gene fusions **(E)** can result in the generation of missense and frameshift neoantigens. Splicing aberrations such as the retention of cancer-specific exons **(C)** and introns **(D)** can also lead to frameshift neoantigens. Epigenetic changes can alter the expression levels of immunogenic genomically-encoded proteins **(F)** including viral proteins from integrated chronically-infected cells (EBV, HPV), cancer-testis antigens (e.g., MAGE-A4) and proteins derived from LINE and HERV elements. Post-transcriptional changes **(G)**, including translation of upstream open reading frames (uORF), stop codon readthrough and protein modifications, such as methylation, phosphorylation and acetylation, can generate tumor specific neoantigens as well.

Neoantigens arise from multiple genetic and epigenetic aberrations (Figure 2). Well-characterized sources of neoantigens are somatic missense and indel mutations, or other genomic rearrangements, such as gene fusions. Frameshift neoantigens may prove to be more immunogenic than missense ones due to the lack of similarity to sequences in the human coding genome and are currently under active investigation (77, 78). Neoantigens derived from gene fusions have recently passed the immunogenicity test (12), and may be of special significance when mutational burden is low. Correct detection of somatic mutations is essential to identify neoantigens incorporated within these alterations (17, 79, 80). Neoantigens can also arise from transcriptome-based aberrations, including cancer-specific gene overexpression, alternative exon splicing, intron retention, premature transcription ending, readthrough the stop-codon by ribosomes and from upstream open reading frames (uORF) (Figure 2). Virus-induced cancers, e.g., HPV+, EBV+, generate strong immune responses due to presentation of viral antigens (81) and as such can be considered as cancer-specific antigens. Transcript-specific changes in exon usage (82, 83), intron retention (84), and transcription end usage were recently shown to produce cancer-specific neoantigens. Translation-based neoantigens, originating from uORF regions, cryptic short ORFs in non-coding RNAs still await their discovery on a pan-cancer level. Whole genome sequencing, deep RNAseq gene expression analysis, whole-cell and MHC-eluate mass-spectrometry will be necessary for a determination of the complete landscape of such neoantigens (85). Finally, cancer-specific post-translational protein modifications, e.g., phosphorylation, acetylation, methylation, citrullination, and etc., can be a potential source of neoantigens as well (86, 87). Aberrant over activity of protein kinases, histone acetylases, and methylases is well-known in multiple cancers. This can result in frequent modifications of non-natural protein targets or cancer-specific proteins, which may in turn produce immunogenic, tumor-specific neoantigens (88, 89) (Figure 2). It is important to point out that T cells with the capacity to recognize these modified antigens likely have escaped central tolerance and thus represent a large pool of T cell clones that could be harnessed to attack cancer cells. Technological advances in mass-spectrometry peptide detection from cancer MHC-I eluates will be essential for neoantigen discovery of this class (90).

## On A Computational Hunt for Neoantigens

### Somatic Mutation Calling

Despite the broad range of potential sources of neoantigens in cancer cells, the process of selection of genomically encoded antigens that are of immunological significance remains to be well-established. Many computational pipelines have been developed to predict neoantigens from cancer genomes (91, 92). A joint effort referred to as the Tumor Neoantigen Selection Alliance (TESLA; supported by the Parker Institute for Cancer Immunotherapy and the Cancer Research Institute) to find the right predictive algorithms for targeting neoantigens (based upon NSVs) through large scale validation is ongoing. At this time, a “typical” neoantigen pipeline includes the following steps:

- Whole exome or genome sequencing (WES or WGS) of tumor and matched normal DNA samples by Illumina short read sequencing platform.
- Quality control of sequencing reads.
- Alignment to the reference genome.
- Base quality recalibration and indel realignment.
- Comparison of normal and tumor alignments to call somatic mutations.
- Conversion of coding DNA somatic mutations to corresponding mutated peptide sequences.
- HLA-allele typing.
- Assessment of HLA-allele and mutated epitope (9–11mer) affinity to call neoantigens.
- Expression analysis of putative neoantigens, e.g., RNAseq, when possible.

**Figure 3:**
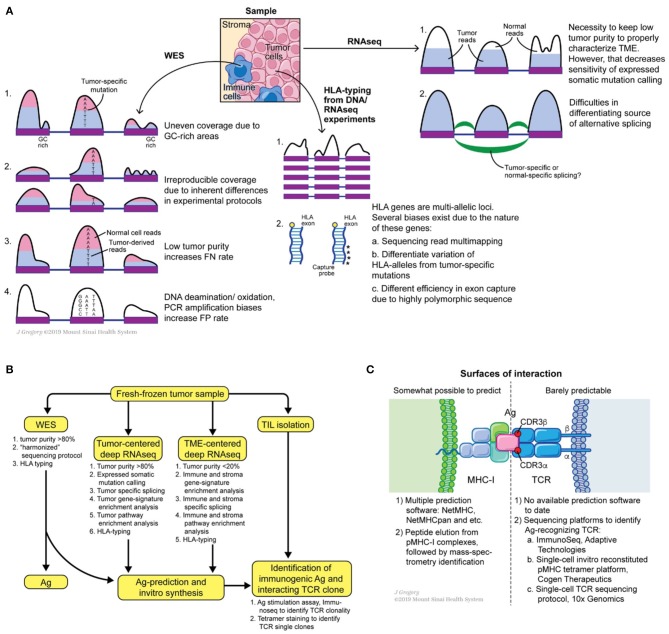
Potential sources of somatic mutation irreproducibility **(A)**, suggested experimental design for comprehensive annotation of tumor mutational burden and tumor microenvironment, **(B)** and possible roads for innovation at the MHC-I antigen TCR interfaces **(C)**.

Multiple tools exist to check the quality of sequencing reads with the most commonly used being FastQC (93, 94). Alternative tools are included in the Genome Analysis Toolkit (GATK) bundle. To perform read alignment, Novoalign (95), BWA (96), bowtie (97), STAR (98) are the most favored aligners. For a typical WES (or WGS) dataset BWA is a commonly used aligner. Base quality recalibration and indel realignment around clusters of putative somatic mutations are both integral tools of GATK and have been shown to reduce the false positive rates of mutation calling (99). Collectively, these “pre-processing” steps output aligned, cleaned, equilibrated ^*^.bam files of tumor and matched normal samples. These matched datasets are fed to a combination of mutation callers to predict somatic mutations in tumor samples. A wide range of somatic mutation callers exist to date, such as Mutect (100), Varscan2 (101), VarDict (102), SomaticSniper (103), Strelka, and FastD (104). Many comparative studies have been performed to call mutations (105–108) (Figure 3A). Some key observations are noted below.

- A combination of multiple algorithms vs. a single mutation caller significantly lowers the false positive rate (108–110).
- Calling somatic mutations from additional sequencing such as of RNAseq of the same tumor sample and determining overlap may help to reduce the false positive rate. However, it may increase the rate of false negatives due to transient gene expression and variable read coverage (111).
- PCR-free WES protocols [KAPA HyperPrep Kit (112)] produce less bias in tumor allele frequencies but achieve it at the expense of reduced total genome loci coverage and of lowered total power of somatic mutation calling (109).
- Exome capture kits (Agilent SureSelect, NimbleGen SeqCap, Illumina TrueSeq, and Illumina Nextera) introduce sequencing coverage biases due to differences in capture probes. This makes it potentially hard to compare final mutation calls obtained from different WES kits of the same sample of DNA, resulting in increased false negative rates for somatic mutation calls (113–116).
- Sequencing read coverage drops significantly in GC-rich regions, decreasing the sensitivity of tumor allele detection in these loci. Correcting for GC-bias may help to rescue certain mutations and improve tumor allele frequency estimations (114).
- Maintaining high tumor purity of the sample before DNA sequencing is essential. High levels of normal DNA “contamination” decreases sensitivity of tumor mutation calling (105, 109, 114).
- The quality of the sample is important, e.g., fresh tissue samples are better than FFPE. It is highly advisable to avoid excessive sample handling known to introduce random DNA mutations, e.g., adenine/cytosine deamination, guanine oxidation, which can impact the results of Illumina sequencing. Otherwise the somatic mutation false positive rates increase (105, 109).

Overall, using fresh or **fresh-frozen** samples with **high tumor purity** (**>80%**), sticking to one **WES protocol**, introducing a **low** number of **PCR cycles**, following **GATK pre-processing** recommendations, and applying **several somatic mutation callers** can benefit the generation of a reproducible, “harmonized” lists of somatic mutations (117). Calling somatic mutations from **RNAseq** of samples with **high tumor content** (**>80%**) can further refine the list of **expressed** mutations. Consensus on unified somatic calling pipelines will be essential to improve the overall prediction of neoantigens and detection of the shared ones.

### HLA-Allele Typing

The next step in a neoantigen calling pipeline is HLA-allele typing. CD8+ T cells see antigens presented on the MHC-I complex, which is composed of conserved β_2_-microglobulin and a variable α-chain. The latter subunit is highly polymorphic and encoded within the HLA gene, which is represented by three loci on human chromosome 6: HLA-A, HLA-B, and HLA-C. Thus, HLA allele assignment consists of the gene name (A, B, or C) followed by a set of digits separated by colons: the first two digits specify serological activity (A^*^01, B^*^03, etc.) and the second two digits indicate protein sequence (A^*^01:05, B^*^03:05, etc.). Due to the high level of polymorphism of each gene, precise HLA-allele typing at protein level resolution from WES and RNAseq reads is a complicated task (118). Multiple tools were developed to address this problem such as PHLAT (119), seq2HLA (120), Optitype (121), Polysolver (122), HLAMatchmaker (123), HLAreporter (124), HLAforest (125), HLAminer (126), xHLA (127). Each tool differs in its performance, utilized set of input parameters and analyzed sequencing dataset (RNAseq or DNAseq). Comparative studies were performed and showed that Optitype has both greater specificity and selectivity (128). However, it is important to keep in mind that the quality of WES/RNAseq is critical for any successful HLA typing. Indeed, due to the highly polymorphic nature of HLA genes, WES capture kits vary in the capturing efficiency of DNA from those regions (Figure 3A). This technical variability in capturing clearly affects downstream results in allele determination. Thus, careful examination of WES/RNAseq read coverage in HLA gene regions is imperative for making optimal predictions.

### Prediction of Neoantigen HLA-Allele Interactions

In the final step, the researcher performs predictions of tumor antigenic epitope- HLA-allele interactions to identify neoantigens from the total pool of mutated peptides. Several tools and programs which are undergoing constant modification, are dedicated to this problem; NetMHC-pan being the one most widely used. NetMHC utilizes a combination of several artificial neural-networks (ANNs) to predict peptide affinity to selected HLA alleles. Initially NetMHC was trained on viral antigens from IEDB [https://www.iedb.org, (129–134)], therefore rendering a bias toward the selection of viral-like epitopes. Despite its general popularity, users should always keep in mind the biases these classification methods can introduce. For example, viral epitopes were originally described for the most frequent HLA-alleles, e.g., HLA^*^A02:01, HLA^*^B07:02. Thus, netMHC based predictions for tumor epitopes are a priori better for highly frequent HLA-alleles than for low-frequency HLA-alleles. One way to overcome this issue is to improve predictions by training the algorithm on peptides eluted from MHC complexes of mono-allelic cancer cell lines and identified by mass-spectrometry analysis (135). However, mass-spectrometry itself has limited ability to detect all possible eluted antigens, thus the false negative rate can be high (90, 136, 137). Data from mass spectrometry analyses indicates that only a small fraction of neoepitopes is presented on the cell surface, likely due to a combination of such systematic biases and real biophysical effects in the processing machinery (138–140). Taken together, there is an urgent need for novel, unbiased methods to generate MHC-I complexes for every HLA-allele with broadly diversified antigen sequences in order to design novel classification tools (Figure 3C).

Apart from class I epitopes, class II restricted neoantigens are receiving increased interest. Class II neoantigens are those epitopes presented by the MHC-II complex and recognized by CD4+ T cells. Despite the recognition that MHC-II is significant for tumor neoantigen presentation and priming of CD4+ T cells (141) and for immunotherapy outcomes (142), the accuracy and precision of MHC-II epitope predictions are poor when compared to class I (143). The main difficulties with designing such classification tools are associated with the “openness” of the peptide-binding groove of HLA class II, which permits binding of a highly degenerate set of peptides, and therefore increasing the size of datasets needed for accurate machine learning-based model training. However, these obstacles provide an opportunity for more creative efforts to develop algorithms to predict such neoantigens.

### Identification of Immunogenic Neoantigen-Reactive T Cells

Not every neoantigen presented on MHC-I complexes will have the capacity to induce CD8+ T cell responses (79). What defines neoantigen immunogenicity? Conventionally, an immunogenic neoantigen must prime and stimulate T cells efficiently. This occurs through (i) interaction of the neoantigen-MHC-I complex with a TCR on one or several T cell clones, and (ii) induction of T cell priming. This process generally results in either TNF-α, IFN-γ, or double TNF-α and IFN-γ cytokine responses, IL-2 release and T cell proliferation, and the acquisition of cytolytic activity in the case of CD8+ T cells. As reviewed above, vaccination has led to the priming and expansion of neoantigen-specific T cells in humans. These responses can be enumerated through assays which measure production of cytokines upon re-exposure to peptides (through ELISA type assays or intracellular staining) or binding to synthetic tetrameric or dextrameic complexes of peptide-MHC (pMHC) molecules. The latter method relies on *in vitro* folding of the MHC-I complex (144, 145) with peptide or UV-cleavable substrate (146) which is later exchanged for the peptide of interest (147).

Neoantigen-specific T cells with effector function have been identified within PBMC following vaccination or even after spontaneous induction (148), tumor infiltrating lymphocytes (149) and can even be differentiated from progenitors through *in vitro* priming approaches (150). A concerted effort is being made to expand potent neoantigen-reactive T cells for the purpose of adoptive cell therapy or to identify high avidity neoantigen-reactive TCRs which can be modified and transduced into a primary T cells. For example, to overcome thymic negative selection, which decreases TCR diversity *in vivo* (151), humanized mice can be used to select the most-optimal neoantigen-reactive TCRs (152). Tetramer-purified, neoantigen-reactive T cell clones can also be expanded from these sources or human blood or TILs in single-cell fashion and their TCRs sequenced. The selected TCRs can be used for recombinant TCR reconstitution (153) and characterization *in vitro*, for additional modification to improve TCR avidity and stability (129, 130) and then adapted for adoptive T cell transfer using a cancer patient's own T cells (131). In this regard tetramer staining can be applied to identify neoantigen-specific TCRs in a high-throughput manner (132). One discovery platform generates *in vitro* translated, DNA-barcoded pMHC complexes from a chemically synthesized DNA library (133). Once tetramer-positive T cells are purified, their interacting TCRs and DNA-barcoded antigens are identified through single-cell sequencing. Moreover, the same platform can be repurposed to characterize all possible peptide specificities for each HLA-allele of MHC-I and MHC-II complexes. Indeed, the ability to (i) start from a randomized DNA library of putative epitopes and (ii) characterize folding potential of produced pMHC complexes in large scale could yield invaluable information to train novel classification algorithms. Despite the obvious advantage of tetramer staining in identifying neoantigen-reactive T cells, this tool provides limited information on the functional status of purified T cells and their cytotoxic capacity (134). The recent development of T-scan screening technology holds promise to overcome this issue (154). Likewise, a recently developed method referred to as imPACT Isolation Technology identifies pre-existing T cell clones that recognize tumor neoantigens (155). Such approaches lay the foundation for multi-group collaborations to synthesize neoantigen-specific T cells for personalized adoptive T cell therapies (155).

Collectively, the identification of immunogenic neoantigens is a multi-step process that requires significant time, cost and labor to accomplish. Personalized neoantigen-based immunotherapies suffer from such drawbacks, sometimes requiring up to 3 months to manufacture the a short list of “best” candidates (156). A potential solution to this pipeline problem is to target shared neoantigens, that are highly recurrent, clonal, and broadly immunogenic across cancer patients. However, whether such immunogenic shared antigens are sufficiently available across broad cancer types remains to be determined. Prioritizing such antigens whenever possible is important, as any “off-the shelf” strategies that can be developed will significantly reduce the cost and increase the efficiency of neoantigen-specific cancer immunotherapies.

## Concluding Remarks

We review the available tools for the computational prediction and experimental validation of tumor-associated neoantigens, discussing approaches for somatic mutation detection, HLA allele typing, and prediction of peptide-MHC interactions. We have made an effort to highlight the biases associated with particular approaches and suggest possible ways to minimize their influence. We also outline technologies for identifying immunogenic neoantigens. Future developments that could improve these strategies are suggested in Figure 3. Firstly, harmonization of somatic mutation calling can improve reproducibility across different platforms and sequencing centers. Secondly, *in vitro* assays for folding and characterization of pMHC complexes starting from randomized peptide libraries can improve existing prediction tools. Applying the same approach for peptide-MHC-II complexes may also improve MHC-II classification tools (157). Finally, single-cell identification of TCR-antigen interacting pairs will provide information on the principles of TCR-neoantigen interactions, making it possible to develop predictive methods for this type of interaction (158). The latter will be an invaluable tool for immunogenic neoantigen selection for vaccine designs, refining immunotherapy outcome predictions, or selecting the most avid TCR for adoptive recombinant T cell therapies. We believe the field of neoantigen-based immunotherapies of cancer is undergoing a major renaissance. Equipped with powerful sequencing technologies, sensitive computational tools for neoantigen discovery and efficient high-throughput platforms for characterization of their immunogenicity, scientists will have the potential to bring novel disruptive immunotherapies to the clinic to definitely improve outcomes of cancer patients.

## Author Contributions

VR wrote the manuscript. VR, BG, and NB reviewed and revised the manuscript.

### Conflict of Interest

NB receives research support or reagents from Novocure, Celldex, the Ludwig Institute for Cell Research, Genentech, Oncovir, and Regeneron, and is on the advisory boards of Neon, Tempest, Checkpoint Sciences, Curevac, Primevax, Novartis, Array BioPharma, Roche, and Avidea. NB receives grant support from and serves on the advisory board of the Parker Institute for Cancer Immunotherapy. The remaining authors declare that the research was conducted in the absence of any commercial or financial relationships that could be construed as a potential conflict of interest.
